# Rapid genotype “independent” *Zea mays* L. (maize) transformation via direct somatic embryogenesis

**DOI:** 10.1007/s11627-018-9905-2

**Published:** 2018-04-30

**Authors:** Keith Lowe, Mauricio La Rota, George Hoerster, Craig Hastings, Ning Wang, Mark Chamberlin, Emily Wu, Todd Jones, William Gordon-Kamm

**Affiliations:** DuPont Pioneer, Johnston, IA USA

**Keywords:** Maize transformation, Direct embryogenesis, Wuschel, BabyBoom

## Abstract

Constitutive expression of the *Zea mays* L. (maize) morphogenic transcription factors *Baby Boom* (*Bbm*) and *Wuschel2* (*Wus2*) in maize can not only greatly increase transformation efficiency but can also induce phenotypic abnormalities and sterility. In an effort to alleviate the pleiotropic effects of constitutive expression, a genome wide search was undertaken to find suitable maize promoters to drive tissue and timing-specific expression of the transformation enhancing genes *Bbm* and *Wus2*. A promoter from a maize phospholipid transferase protein gene (*Zm*-*PLTP*_*pro*_) was identified based on its expression in leaves, embryos, and callus while being downregulated in roots, meristems, and reproductive tissues. When *Zm-PLTP*_*pro*_ driving *Bbm* was transformed into immature maize embryos along with a *Wus2* expression cassette driven by the nopaline synthase promoter (*Nos*_*pro*_::*Wus2*) abundant somatic embryos rapidly formed on the scutella. These embryos were individual and uniformly transformed and could be directly germinated into plants without a callus phase. Transformed plants could be sent to the greenhouse in as little as 1 mo and regenerated plants matched the seed-derived phenotype for the inbred and were fertile. However, T1 seed from these plants had poor germination. Replacing *Nos*_*pro*_ with a maize auxin-inducible promoter (*Zm-Axig1*_*pro*_) in combination with *Zm-PLTP*_*pro*_::*Bbm*, allowed healthy, fertile plants to be regenerated. Single-copy T1 seed germinated normally and had a predominantly wild-type inbred phenotype. For maize, this callus-free transformation process has worked in all inbred lines tested.

## Introduction

Since the discovery that immature maize embryos could be used to initiate regenerable embryogenic callus (Green and Phillips 1975), almost all high throughput transformation systems in cereals including maize have required callus formation and selection prior to plant regeneration (see Ji *et al.* 2013 for review). Immature embryos have been the predominant explant for transformation but some success has been obtained using particle bombardment of alternative explants such as meristems and leaves (Wang *et al.* 2009). Typically, immature embryos are transformed and embryogenic callus is induced and bulked up under selective pressure to obtain a mass of clonal tissue that can then be regenerated into plants. This process is both labor- and time-intensive taking from 87 to 140 d from initiation of transformation to sending plants to the greenhouse (Wang 2005; Ishida *et al.* 2007).

Lowe *et al.* (2016) have recently described a transformation method that uses the maize transcription factors Baby Boom (*Bbm*) and Wuschel2 (*Wus2*) to stimulate the proliferation of transformed cells. In this case, cells were directly induced to form callus by the transgenes rather than transforming cells that had been induced to divide by conventional tissue culture methods. Unfortunately, using this method, pleiotropic effects were observed in transgenic plants making excision of *Bbm* and *Wus2* necessary to obtain normal transgenic plants. To alleviate this problem, Lowe *et al.* (2016) used a desiccation inducible promoter, Rab17_*pro*_, to control CRE-mediated excision, a method that was compatible with embryogenic callus culture. However, the Lowe *et al.* (2016) method still required 3 mo of callus selection before transferring callus to dry filter papers for 3 d to induce excision.

In the present method, described below, somatic embryo formation occurred within the first week after *Agrobacterium tumefaciens* infection, and then the somatic embryos were moved onto medium containing low hormone levels to stimulate further somatic embryo maturation and germination. Within this rapid timeframe, the RAB17_*pro*_ was too slow to induce excision and the desiccation treatment was deleterious to the development of small somatic embryos. Thus, another method of controlling WUS and BBM expression was required.

Another approach with the potential to obviate pleiotropic effects of *Bbm* and *Wus2* was to find promoters that could drive transgene expression in young leaves, embryos, and callus while being off in roots, meristems, and reproductive tissues. A survey of Illumina® gene expression data from over 65,000 maize gene models identified only one gene that fully exhibited strong expression in embryos and leaves, while exhibiting low expression in the ear, tassel, and tassel meristem, and with no detectable expression in roots. The promoter from this gene (a phospholipid transferase protein, *Zm-PLTP*_*pro*_), was then used to drive *Bbm* expression *(Zm-PLTP*_*pro*_::*Bbm).* Surprisingly, when *Zm-PLTP*_*pro*_::*Bbm* in combination with *Nos*_*pro*_::*Wus2* were transformed into immature zygotic embryos, uniformly transformed somatic embryos rapidly formed on the scutellar epithelium. These somatic embryos could be germinated directly into healthy fertile plants without any callus formation. However, the T1 seed from the transgenic plants containing *Nos*_*pro*_::*Wus2* had inconsistent germination. This problem was overcome by using a maize auxin-inducible promoter Zm-*Axig1* (Garnaat *et al.* 2002) to drive *Wus2* expression. Transformation of immature embryos using the combination of *Zm*-*PLTP*_*pro*_::*Bbm* and *Zm*-*Axig1*_*pro*_::*Wus2* resulted in the rapid proliferation of somatic embryos from the scutellar epithelium. These embryos were germinated directly into fertile plants without excision of the morphogenic genes. This process is very rapid and transition stage somatic embryos were visible just 8 d after transformation of immature embryos, growing at approximately the same rate as their zygotic counterparts. To date, this callus-free transformation process in maize immature embryos has worked in all maize genotypes tested and is both efficient, and fast.

## Materials and Methods

### Plant material

Three public inbred lines, Fast-Flowering Mini-Maize (FFMM line A) which flowers within 25–28 d after planting (see McCaw *et al.* 2016), B73, Mo17, and five DuPont Pioneer inbred lines were used in these experiments, including two stiff-stalk inbred lines (PHH5G and PH1V69) and three non-stiff-stalk inbred lines (PHR03, PH184C, and PH1V5T). One of the DuPont Pioneer inbred lines reported in this article (PHR03) is non-proprietary and publicly available along with B73 and Mo17 from USDA-GRIN (https://www.ars-grin.gov/). The other four DuPont Pioneer inbred lines described in this research are proprietary (PHH5G, PH1V69, PH1V5T, and PH184C). All plants used for source immature embryos were greenhouse-grown.

### *Agrobacterium tumefaciens* strains and vectors

An auxotrophic (THY-) version of *A. tumefaciens* strain LBA4404 containing pVir9, a separate accessory plasmid containing Bo542 virulence genes (Anand *et al.* 2017a, b, GenBank accession# MF788073) was used for all immature embryo transformations (PHP79065, PHP79066, and PHP79094). Table 1 lists all of the molecular components (promoters, genes, 3′ sequences, etc.). The *Axig1* promoter used in this study was modified by the addition of a single tet operator sequence (Gatz *et al.* 1992) placed at the transcription start site with the thought that additional repression would be necessary to obtain normal plants. This was found to be unnecessary and all future vectors were constructed without this operator and no differences in transformation or plant phenotypes were observed.

**Table 1.** Genetic components used to construct expression cassettes within the T-DNAs

Component type	Label	Description	References
Promoters	*Ubi*_pro_	The maize ubiquitin promoter, the 5′ UTR, and the first intron	Christensen *et al.* (1992)
	*Sb-ALS*_pro_	The sorghum ALS promoter	SB-ALS promoter and 5′ UTR, DOE-JGI Sbi v3.1, SBChr04, bases 49239164-49240031. DOE-JGI Sbi v3.1 corresponds to *Sorghum bicolor* BTx623 assembly v3.0.1 and gene annotation v3.1 available from phytozome (http://phytozome.jgi.doe.gov/). Chromosome 4 of Sbi v3.1 is registered as NCBI accessions NC_012873.2 and CM000763.3
	PLTP_pro_	Maize phospholipid transferase promoter	Anand *et al.* (2017a, b) US20170121722
	LTP2_pro_	Barley lipid transferase promoter	Kalla *et al.* (1994)
	*nos*_pro_	The *Agrobacterium*-derived nopaline synthase promoter	An (1986)
	Axig1_pro_	The maize Axig1 promoter	Garnaat *et al.* (2002) WO2002006499
	*rab17*_pro_	The maize *rab17* promoter and 5’ UTR	Busk *et al.* (1997)
3′ Sequences	PINII	The potato proteinase inhibitor II (pinII) 3′ sequence	An *et al.* (1989)
	*Sb-PEPC* 3′	The PepCarboxylase 3′ regulatory sequence from *Sorghum bicolor*	Anand *et al.* (2017a, b) US20170121722
	*Os-T28* 3’	The T28 3′ regulatory sequence from *Oryza sativa*	Bhyri *et al.* (2014) US20140130205 A1
Marker genes	PMI	The phosphomannose isomerase gene from *E. coli*	Negrotto *et al.* (2000)
	HRA	The maize ALS double mutant gene conferring herbicide resistance	Green *et al.* 2009
	moPAT	A maize codon-optimized gene encoding phosphinothricin acetyltransferase	Jayne *et al.* (2000) US6096947
	*ZsYELLOW*	The *ZsYellow*1 N1 gene encoding a yellow fluorescent protein from *Zoanthus* sp.	Matz *et al.* (1999)
	*ZsGREEN*	A gene encoding the *ZsGREEN* protein from *Zoanthus* sp.	Matz *et al.* (1999)
Maize morphogenic genes	*Zm-Wus2*	The maize *Wuschel2* (*Wus2*) gene	Lowe *et al.* (2007) US7256322B2
	*Zm-Bbm*	The maize *Baby boom* gene (*Bbm*)	Gordon-Kamm *et al.* (2005) WO2005075655

Cartoons depicting the arrangement of expression cassettes within the T-DNA of plasmids used in these experiments can be found in Fig. 1. PHP79094 contained three expression cassettes (Fig. 1*A*). The first cassette contained the maize *PLTP* promoter driving *ZsGREEN* (Takare Bio USA; Mountain View, CA), the second contained the maize *Ubi* promoter and intron driving PMI and the third contained the rice Actin promoter driving moPAT. PHP79065 contained four expression cassettes: the first contained the *Nos* promoter driving *Wus2*, the second contained the *Zm-PLTP* promoter driving *Bbm*, the third contained the Sorghum *ALS* promoter driving the maize *Hra* (a double mutant of the maize ALS gene that confers resistance to sulfonylureas and imidazolinones, see Green *et al.* 2009), and the fourth contained the barley *LTP2* promoter driving *ZsYELLOW* (Fig. 1*B*). PHP79066 was identical to PHP79065 except the maize *Axig1* promoter was used in place of the *Nos* promoter to drive *Wus2* (Fig. 1*C*).

**Figure 2.**
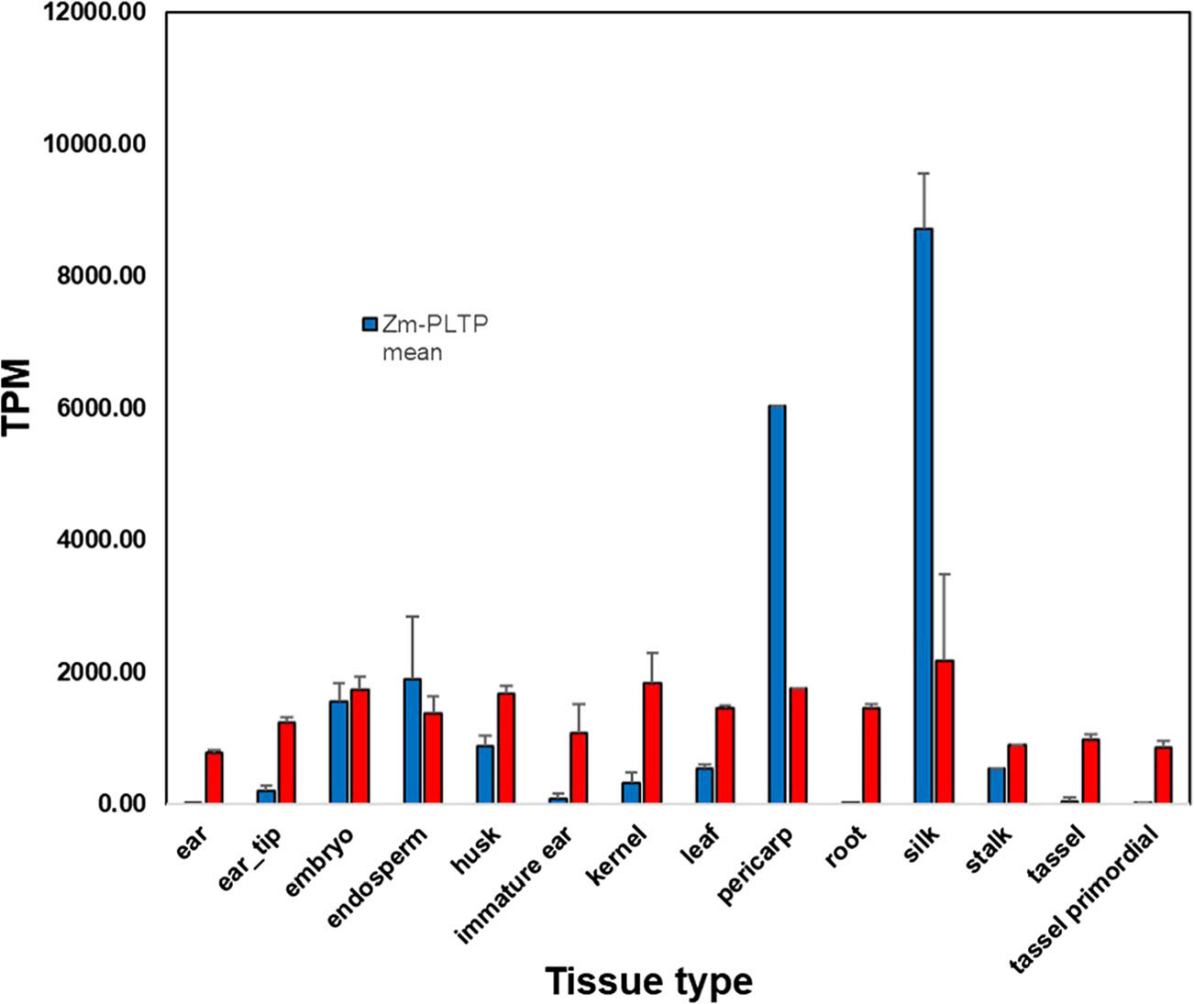
Gene expression profile for *Zm-PLTP* and *Zm-UBI*. Values are the average gene expression across samples grouped by major tissue category (unit: parts per million sampled reads or the numerical equivalent transcripts per million). *Black bars* represent the 95% confidence interval around mean). All samples contained multiple bio-replicates except for pericarp and stalk

Some materials reported in this paper contained the selectable markers moPAT and PMI owned by Bayer Crop Science International AGb (Leverkusen, Germany) and Syngenta International AG (Basel, Switzerland), respectively (see Table 1 and references therein). Pioneer Hi-Bred International, Inc. (Johnston, IA) will provide plasmids to academic investigators for non-commercial research under an applicable material transfer agreement subject to proof of permission from any third-party owners of all or parts of the material and to governmental regulation considerations. Completion of a stewardship plan is also required. Obtaining permission from third parties will be the responsibility of the requestor.

### Culture media used for transformations and plant regeneration

All media recipes are described by Lowe *et al.* (2016). For the rapid methods described above 0.1 mg L^−1^ imazapyr (Sigma-Aldrich®; St. Louis, MO). was added to the somatic embryo maturation and germination steps.

### Immature embryo isolation, *A. tumefaciens*-mediated transformation, and tissue culture

Immature embryos (average length 2 mm isolated from greenhouse-grown plants) were transformed with PHP79094 and selected on mannose as described by Zhao *et al.* (2002), adapted for PMI selection (see also Reed *et al.* 2001). For PHP79065 and PHP79066, embryos were isolated and transformed as described by Lowe *et al.* (2016) but cultured differently. Briefly, transformation was performed as follows: *A. tumefaciens* strain LBA4404 THY- containing one of the above transformation vectors was grown overnight in the dark at 26°C on plates containing YP medium (Ishida *et al.* 1996) and colonies were collected and suspended in PHI-A700 liquid medium. Immature embryos were mixed with *A. tumefaciens* strain LBA4404 THY- (OD = 0.7 at 550 nm) in 700 liquid medium for 5 min and were then removed from the liquid and placed scutellum side up on 710I solid medium (co-cultivation medium) overnight at 21°C in the dark (for PHI-A700, 700, and 710I media, see Zhao *et al.* 2002). The following morning embryos were moved onto 605 J somatic embryo induction medium and cultured in dark at 28°C. After 6–7 d on 605 J medium, the embryos were moved onto 289O maturation medium containing 0.1 mg L^−1^ imazapyr. After 2 wk on 289O medium, embryos were moved to 13158 medium also containing imazapyr for germination and placed under GE Ecolux® (General Electric; Boston, MA) fluorescent lights G (60 μmol m^−2^ s^−1^) with a 16-h photoperiod at 26°C.

Transformation frequency was defined as the number of treated immature embryos that produced imazapyr-resistant T0 plants (PHP79065 or PHP79066). Imazapyr selection was maintained during somatic embryo germination and plantlet production to preclude escapes. Once shoots and roots had been established, plantlets were transferred to pots in a fully automated greenhouse using proprietary protocols (however, procedures for growing maize under greenhouse conditions are well established; see https://docs.lib.purdue.edu/pmcg/).

### Molecular analysis

All molecular analysis was done as described by Lowe *et al.* (2016) for polymerase chain reaction (PCR) analysis and Zastrow-Hayes *et al.* (2015) for Southern-by-Sequencing.

### Fluorescence microscopy and histology, sectioning, and microscopy

Fluorescence microscopy was performed as described by Lowe *et al.* (2016). To further illustrate the morphology of the rapidly forming somatic embryos, zygotic embryos with protruding nascent somatic embryos were sampled at 2, 4, 6, and 7 d after the beginning of *A. tumefaciens* infection and fixed in 2.5% (*v/v*) EM-grade glutaraldehyde in 100 mM phosphate buffer (pH 7.0) at 27°C with rotation at 100 rpm (VWR Standard Orbital Shaker, Model 3500; Radnor, PA) for 4 h. After washing with 100 mM phosphate buffer (pH 7.0) three times for 15 min each, the samples were dehydrated in a step-wise fashion; 1–2 h in 70% (*v/v*) EtOH, 1–2 h in 80% (*v/v*) EtOH, 1–2 h in 95% (*v/v*) EtOH, 1–2 h in 100% EtOH, and 2 h in 100% EtOH. The tissue was then infiltrated with activated Technovit® 7100 glycolmethacrylate (Heraeus Kulzer GMBH; Hanau, Germany), following the manufacturer’s recommendation, for 2 h, and again with refreshed activated Technovit® 7100 overnight. The infiltrated tissue samples were placed in molds, and the Technovit® 7100 was polymerized by the addition of 1 mL Hardener II into 15 mL of activated Technovit® 7100. The molds were placed under house vacuum of approximately 685.5 mmHg in a vacuum desiccator chamber and allowed to polymerize overnight. Semi-thin sections of 2 μm were cut with a Leica 2065 microtome (Leica Biosystems; Wetzlar, Germany), placed on drops of distilled water on glass slides and dried on a heating plate at 45°C. Sections were stained with periodic acid-Schiff reagent (Sigma-Aldrich®) according to instructions and were then counter-stained with 0.5% (*w/v*) Naphthol blue black in 7% (*v/v*) acetic acid solution for 5 min and washed again in distilled water. The sections were then mounted in Permount™ (Thermo-Fisher Scientific®; Waltham, MA) with glass coverslips and observed.

## Results

### Survey of Illumina® RNAseq gene expression data and identification of *Zm-PLTP*

An internal proprietary maize transcriptome atlas consisting of approximately 65,000 gene models from DuPont Pioneer’s maize gene dataset sequenced was surveyed with the Illumina® RNAseq protocol. The transcriptome atlas was sourced from over 500 mRNA samples including an average of four bio-replicates from most major maize tissues and developmental stages ranging from VE to V19; R1 to R5; and multiple embryo parts (leaf emerging, leaf production, and reproductive growth stages in the Growth Stage Ontology; Ritchie *et al.* 1986; Pujar *et al.* 2006). The RNA Sequencing by Expectation Maximization method (RSEM; Li and Dewey 2011) was used as the metric for the quantification of gene expression in transcripts per million reads observed normalized units (TPM) with each gene model’s TPM estimate summarizing the expression value for all its measurable isoforms in a way that is relative to the total number of sequences in a particular sample’s library.

In order to simplify gene expression profile scanning, the data matrix was reduced by calculating average RSEM expressions per gene for all biological replicated samples, thus reducing the dataset to about 130 tissues per development stage combinations and by filtering genes with very high variance within sets of biological replicates. Python scripts calculated a series of metrics per gene that allowed detection of expression outliers, including internal implementation of the Shannon’s entropy for information content (Hs) and the Minimum Akaike’s Information Criterion (MAICE, AIC) as defined in the original ROKU method and improved on by the ROKU-SPM method https://www.rdocumentation.org/packages/TCC/versions/1.12.1/topics/ROKU (Kadota *et al.* 2003, 2006, 2007; Guo *et al.* 2013) and the resulting matrix loaded into TIBCO Spotfire® 7.0 software (TIBCO® Software Inc.; Palo Alto, CA). The new metrics were used to filter and retain genes for which expression values across all root and root-tissue samples were effectively zero (based on outlier detection). Further filtering retained genes with overall “medium to high” stable expression values across other tissues of interest (leaf at multiple stages; embryo and sub-tissues) reducing the set to only 11 gene candidates. Two candidates were shortlisted for comparison of their promoter with the standard transformation *Zm-UBI* promoter. Of these two, only *Zm-PLTP* had both high expression in embryo tissues and abundant matching ESTs sequenced in independent callus libraries. The expression profile for this gene and that of *Zm-UBI* are shown in Fig. 2 for comparison.

**Figure 1.**
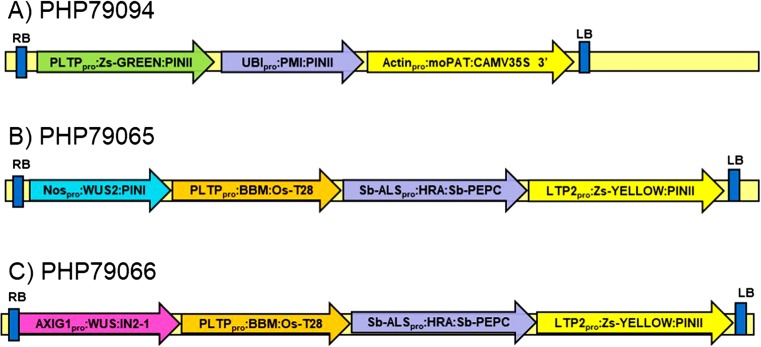
T-DNA composition for vectors used in corn transformation experiments. *A* depicts the *Zm-PLTP*_*PRO*_::*ZsGREEN* reporter construct, while *B* and *C* depict the arrangement of *Wus2*, *Bbm*, *Hra*, and *ZsYELLOW* expression cassettes in PHP79065 and PHP79066

### Expression of *Zm-PLTPpro*::*ZsGREEN* in maize

**Figure 3.**
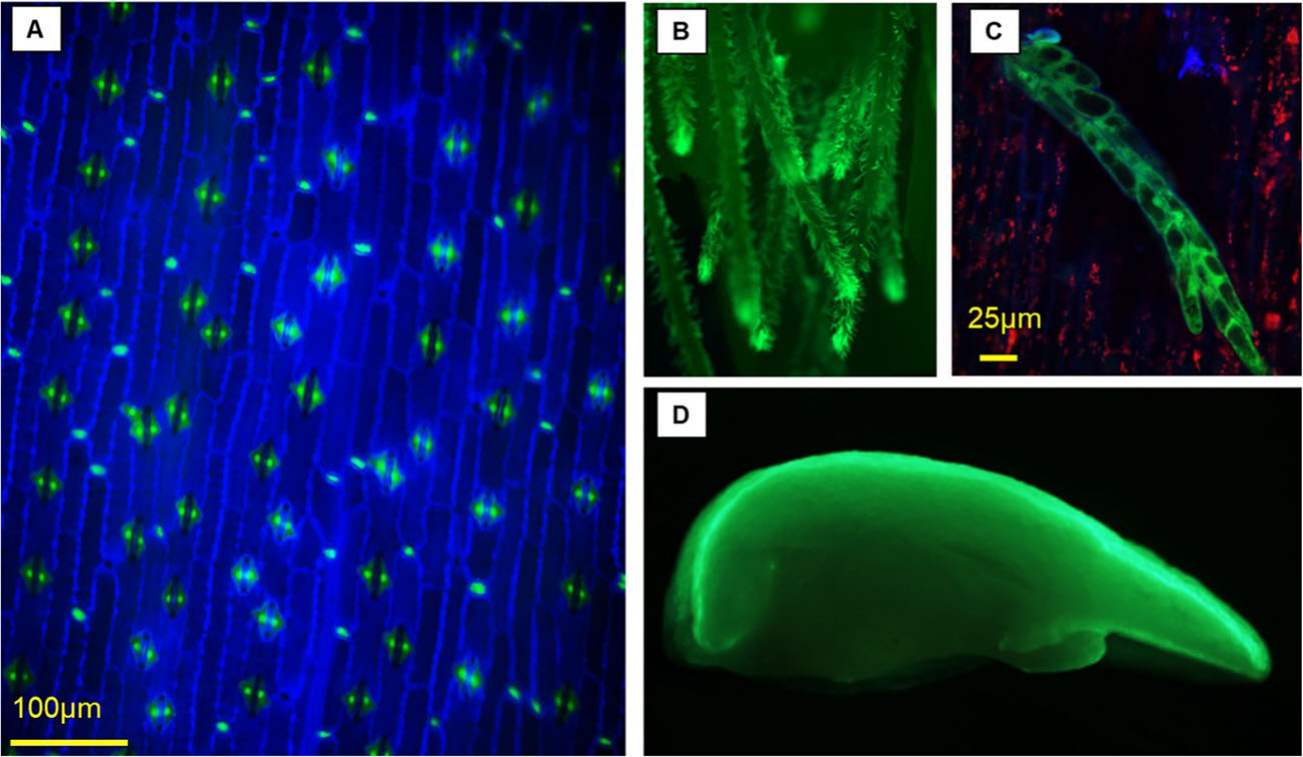
*Zm-PLTP*_*PRO*_*::ZsGREEN* expression in *Zea mays* L. in the pairs of subsidiary cells adjacent to the guard cells, and in the small, single cork cells observed between the elongated epidermal cells in leaves (*A*), in silk hairs (*B* and *C*), and in across-section of an immature T1 embryo (*D*)

A 1062-bp fragment upstream of the *Zm-PLTP* coding sequence containing the *PLTP* promoter and 5′ UTR was synthesized by GenScript and designated herein as *Zm-PLTP*_*pro*_. Immature embryos from the maize inbred PH184C were transformed with PHP79094 (Fig. 1*A*), a plasmid containing *Zm-PLTP*_*pro*_::*ZsGREEN*, *UBI*_*pro*_::*PMI*, and *Os-Actin*_*pro*_::*moPAT*, using conventional callus transformation procedures. qPCR was used to identify single-copy plantlets and these were sent to the greenhouse for observations. *ZsGREEN* expression was observed in callus prior to plant regeneration (not shown) and was also observed in leaf subsidiary and cork cells in T0 plants (Fig. 3*A*), as well as in silks of the T0 plants (Fig. 3*B*, *C*). T1 embryos at 14 d after pollination (DAP) were rescued and strong *ZsGREEN* expression was observed in the scutellar epithelial layer after being placed on culture medium (Fig. 3*D*). The visual expression patterns were consistent with the Illumina® expression data.

### *Zm-PLTPpro*::*Bbm* with either *Nospro*::*Wus2* or *Zm-Axig1pro*::*Wus2* induced somatic embryo formation in immature zygotic embryos

**Figure 4.**
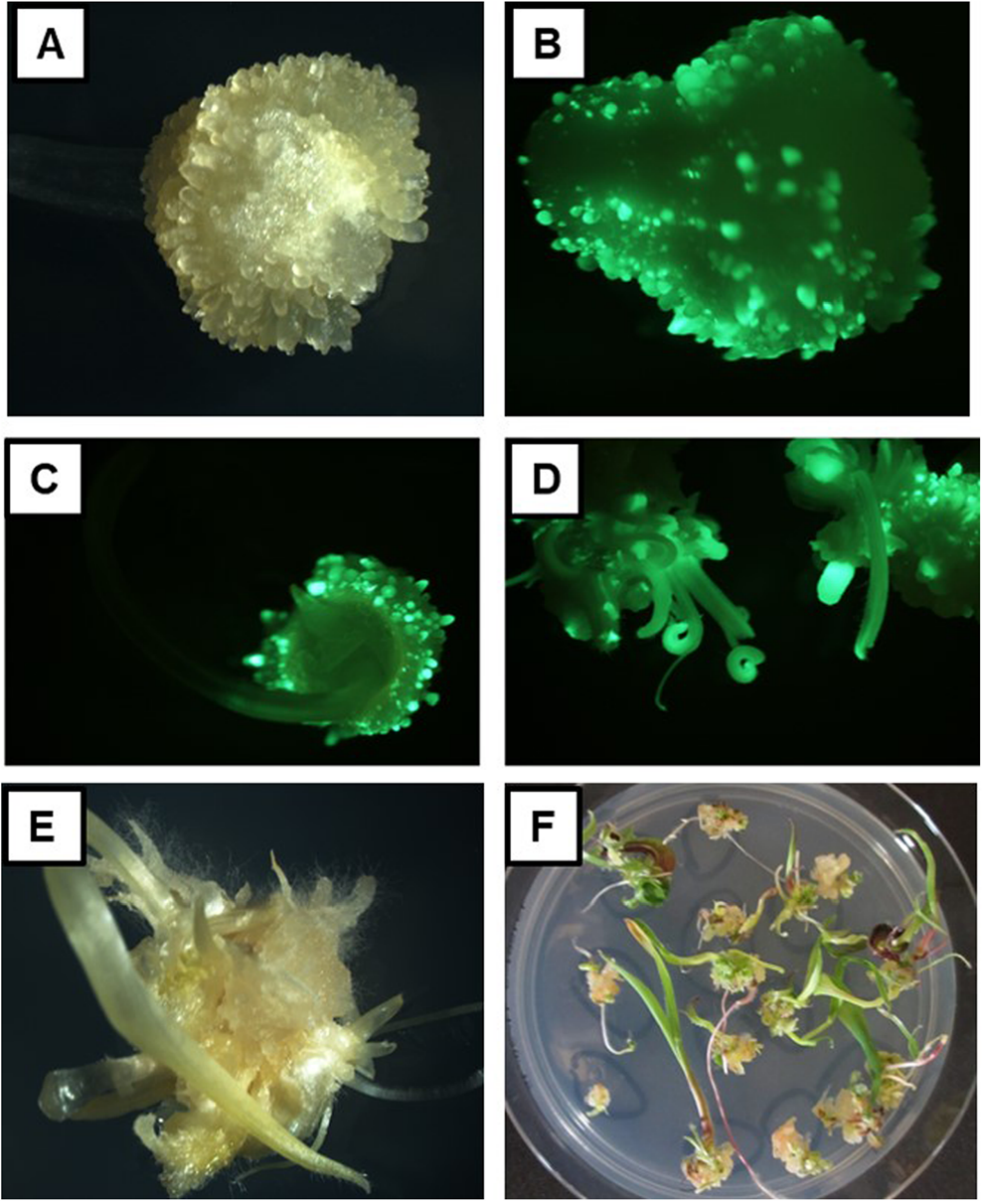
Rapid development of single somatic embryos of *Zea may* L. on the surface of recently transformed zygotic immature maize embryos shown under bright field microscopy for Fast-Flowering Mini-Maize at 7 d after infection (*A*). Green fluorescence of somatic embryos at progressive durations after *Agrobacterium tumefaciens* infection are shown for pioneer inbred PH184C (at 4 d (*B*), 7 d (*C*), and 10 d (D) after infection ). *E*, *F* Germinating transgenic plantlets using the public inbred Mo17 at 14 and 24 d (respectively) after *A. tumefaciens* infection showing 1–2 plantlets developing from each of the originally infected immature embryos

Immature zygotic embryos were transformed with PHP79066 (*Zm-Axig1*_*pro*_::*Wus2 + Zm-PLTP*_*pro*_::*Bbm* Fig. 1*C*) or PHP79065 (*Nos*_*pro*_::*Wus2 + Zm-PLTP*_*pro*_::*Bbm* Fig. 1*B*). A modified version similar to PHP79066 was constructed with (*Zm-Axig1*_*pro*_::*Wus2 + Zm-PLTP*_*pro*_::*Bbm + UBI*_*pro*_::*ZsGREEN*) to permit visualization of fluorescence during all stages of somatic embryo development and early plantlet formation (Fig. 4*B*–*D*). After an overnight co-culture at 21°C, the immature embryos were transferred to a cytokinin-free somatic embryo initiation medium containing 0.8 mg L^−1^ 2,4-D and 1.2 mg L^−1^ dicamba (which is an inducer of the *Zm-Axig1*_*pro*_). Within 4 d, the surfaces of almost all of the zygotic embryos were covered with well-formed somatic embryos (Fig. 4*A*, *B*). The production of somatic embryos appeared to be genotype-independent. Based on *ZsGREEN* expression, these embryos were uniformly transformed suggesting they were of single cell origin (Fig. 4*B*–*D*). Somatic embryos were transferred to maturation medium at 7 d after infection (DAI) and rapidly began germinating (Fig. 4*D*, *E*). The germinating plantlets were transferred onto medium with reduced auxin levels (to promote root elongation) 1 wk later (14 DAI) and were ready for transplanting in the greenhouse within 24 DAI (Fig. 4*F*). Following the methods described in Lowe *et al.* (2016), with the modification of using a plasmid containing *Zm-Axig1*_*pro*_::*Wus2 + Zm-PLTP*_*pro*_::*Bbm*, the rapid formation of somatic embryos in alternative explants such as leaf segments or sections from mature seed was not observed. Instead, a brief intervening embryogenic callus phase was observed for 2–3 wk, followed by plant regeneration.

### Anatomy of somatic embryos

**Figure 5.**
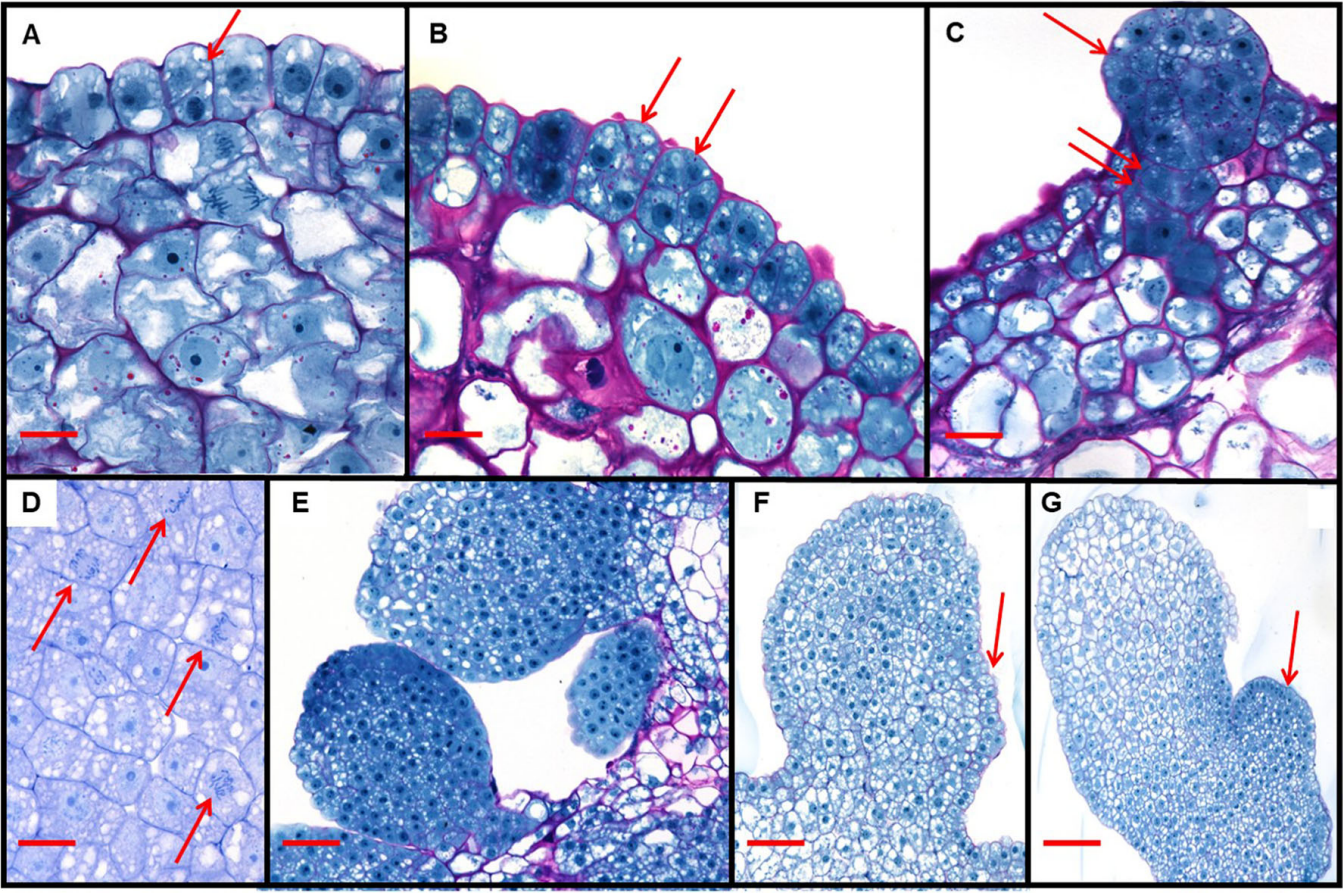
Histology of *Zea mays* L. somatic embryo development after *A. tumefaciens*-mediated transformation of immature embryos with a T-DNA containing the expression cassettes *Zm-Axig1*_*pro*_::*Wus2* plus *Zm-PLTP*_*pro*_::*Bbm*. The earliest morphological change observed were transverse or oblique cell divisions (arrow, *A*), continuing to divide and become multicellular (arrows, *B*) and growing into early globular pro-embryos (arrow, *C*) with some being subtended by what appeared to be a suspensor (double arrow, *C*). In the developing somatic embryos, multiple mitotic figures were often observed in the same cross-section (arrows, *D*). In *E*, multiple independent somatic embryos were observed in close proximity, and as somatic embryos continued to develop, the embryonic meristem could be observed to develop (arrows, *F*, *G*). *Scale bar*: *A* 12 μm; *B*–*D* 25 μm; *E*, *F* 50 μm; *G* 100 μm

In order to determine the origin and ontogeny of the morphogenic gene-derived somatic embryos, zygotic embryo explants at 2, 4, 6, and 8 DAI were fixed, embedded, sectioned, and stained for light microscopy. At the stage of development that the zygotic embryos were used for transformation (approximately 2 mm in length), the epidermal layer facing the endosperm had differentiated into a scutellar epithelium, a layer of columnar cells that divide slowly relative to earlier stages of zygotic embryo development. These cells were the target cells for *A. tumefaciens*-mediated transformation. The first physiological indications of *Wus2* and *Bbm* expression were transverse or oblique cell divisions in scutellar epithelium cells (Fig. 5*A*). Occasionally, there was evidence of mitotic activity in cells immediately subjacent to the scutellar epithelium, possibly indicating a response to non-cell autonomous movement of the WUS protein from *Wus2* expressing cells (or other diffusible factors) into non-transgenic neighboring cells. The epithelial cells continued to divide (arrows in Fig. 5*B*) into 4-, 8-, and 16-cell clusters and eventually, by 4 DAI, globular embryos were observed, often with a short suspensor-like structure connecting the embryo to the scutellum of the original zygotic embryo (Fig. 5*C*). The cell division rate of the developing embryos was very high, with an estimated doubling time of approximately 8–10 h (Fig. 5*D*). At 4 DAI, the size of many somatic embryos and the number of cells in these globular embryos was very similar to those observed in 4-d-old zygotic embryos by Kiesselbach (1949). The somatic embryos continued to develop into larger globular-shaped structures (Fig. 5*E*) and finally, by 8 DAI, they developed into embryos with a differentiated epidermal layer that had formed a scutellar notch where the shoot apical meristem would eventually develop (Fig. 5*F*, *G*). The somatic embryos induced by *Wus2* and *Bbm* expression were derived from single scutellar epithelial cells. Subsequent embryonic development closely recapitulated the ontogeny of maize zygotic embryos (Randolph 1936; Kiesselbach 1949) as well as rice somatic embryos derived from the scutellum epithelial layer of mature rice embryos under 2,4-D induction (Jones and Rost 1989). Somatic embryo development also resembled somatic embryogenesis from leaves as described in orchardgrass (Conger *et al.* 1983)

### Direct germination of somatic embryos induced with *Zm-PLTPpro*::*Bbm* and *Zm-Axig1pro*::*Wus2*

Immature embryos were transformed with PHP79066 and incubated overnight on 710I co-cultivation medium. The next day, these embryos were moved to 605 J somatic embryo initiation medium and cultured in the dark for 7 d. After 7 d, the zygotic embryos with rapidly proliferating somatic embryos were moved to 289O maturation medium containing 0.1 mg L^−1^ imazapyr for selection, ending the exposure to exogenous hormones (i.e., 2,4-D and dicamba) at this point. After 2 wk on 289O maturation medium, the zygotic embryos and attached somatic embryos were moved to 13,158 germination medium containing 0.1 mg L^−1^ imazapyr and placed under fluorescent lights. Many of the somatic embryos germinated, and after 2–4 wk, plantlets were transplanted into flats and moved into the greenhouse. Plants were sampled for qPCR and copy number determined for *Wus2*, *Bbm*, and *Hra* (amplifying across the junction of the coding sequences and terminators). Events that were single copy for *Wus2*, *Bbm*, and *Hra* and that were also backbone negative were designated quality events.

### Transformation frequencies

Plant transformation frequencies [(number of T0 plants ÷ number of starting immature embryos) × 100] ranged from 8.7 to 96% based on the number of starting embryos (see Table 2). In a separate set of experiments, larger numbers of immature embryos from three Pioneer inbred lines (PHR03, PHH5G, and PH1V69) were transformed and copy number analysis was determined on individual plantlets (Table 3). For these three inbred lines, transformation frequencies ranged from 9.1 to 62.5%.

**Table 2.** Numbers of independent *Zea mays* L. cv. PHP79066 T0 events produced from various pioneer and public inbred lines. For each inbred, replicates are indicated in separate rows, with each row reflecting immature embryos harvested from a single ear. Transformation Frequency calculated as (number of T0 plants ÷ number of embryos) × 100

Genotype	Number of embryos	Number of T0 plants	Transformation frequency (%)
PHR03	72	32	44
PHR03	41	27	66
PHR03	36	27	75
PHR03	39	30	77
PH184C	188	57	30
PHH5G	75	168	224
PH1V5T	90	26	29
Mo17	133	46	35
Mo17	172	25	15
Mo17	51	16	31
B73	36	18	50
B73	46	4	9
B73	61	8	13
Mini-Maize “A”	105	101	96
Mini-Maize “A”	150	132	88

**Table 3.** Summary data for transformation experiments for the pioneer *Zea may* L. inbred lines PHR03, PHH5G, and PH1V69 after transformation with a T-DNA containing *Zm*-*PLTP*_*pro*_::*Bbm* plus either *Nos*_*pro*_::*Wus2* (PHP79065) or *Zm-Axig1*::*Wus2* (PHP79066), showing the number of immature embryos infected with *Agrobacterium tumefaciens*, the resulting number of T0 plants sampled for PCR, the transformation frequency ((number T0 plants ÷ initial number of Embryo) × 100), the number of plants that were single copy for the transgenes (quality events based on qPCR) and the final frequency of usable quality events (single copy with no backbone relative to the original number of starting immature embryos). For each inbred, replicates are indicated in separate rows, with each row reflecting immature embryos harvested from a single ear

Genotype	Plasmid	Embryo Number	Number of Plants sampled	Transformation Frequency (%)	Plant QE Number	%UQE
PHR03	79065	210	60	28.6	10	4.8
PHR03	79066	200	125	62.5	25	12.5
PHH5G	79065	162	41	25.3	11	6.8
PHH5G	79066	165	15	9.1	6	3.7
PH1V69	79065	148	37	25	9	6
PH1V69	79066	155	47	30.3	14	9

This rapid transformation method successfully produced transgenic T0 plants in immature embryos isolated from all the ears tested (for all eight inbred lines used in the experiments summarized in Tables 2 and 3). However, transformation frequency varied between inbred lines with averages ranging from 9% for one ear from B73 to 224% for one ear from PHH5G (since more than one independent somatic embryo originating from a single *A. tumefaciens*-infected zygotic embryo could germinate to form T0 plants, transformation frequencies above 100% could be observed). Variability was also observed between individual ears from the same inbred (for example in B73 which produced transformation frequencies of 9, 13, and 50% for immature embryos isolated from the three ears tested). For the copy number analysis in T0 plants shown in Table 3, if a plant was single copy for all the transgenes and with no *A. tumefaciens* backbone, it was considered a quality event (QE) relative to the number of T0 plants analyzed, while usable quality event frequencies (UQE%) referred to the frequencies of quality event relative to the original number of starting immature embryos and ranged from 3.64 to 12.5%.

### T0 and progeny analysis

**Figure 6.**
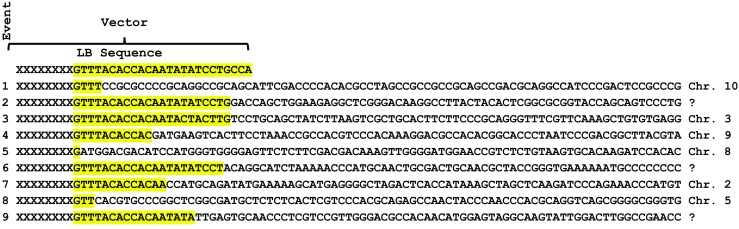
SbS™ data from nine single copy *Zea mays* L. events produced in inbred PHR03 showing the flanking sequences between the left border (*yellow*) and chromosomal DNA. Chromosome assignments are based on B73 models which may not be representative of this inbred. In some cases, the T-DNA insertion integrated into a repetitive region and chromosome location could not be accurately assigned

While all the transgenic events were confirmed by qPCR, production transformation requires higher standards and these events were further characterized to determine the site of integration and to confirm that endogenous genes were not disrupted. To demonstrate integration, Southern-by-Sequencing data (SbS™, see Zastrow-Hayes *et al.* 2015) is shown for the junction region around the left border for nine events that had previously been characterized as single-copy events by qPCR (Fig. 6). All nine events had T-DNA flanking regions that mapped to the B73 genome build and each of the two flanking genomic sequences (two per event) mapped to the same chromosomal location.

T1 progeny segregation was evaluated using qPCR for the cassette components in the T-DNA to determine zygosity in T1 seedlings from four self-pollinated PH1V5T T0 plants and two self-pollinated PHR03 T0 plants. For all six plants that were self-pollinated, the progeny segregated as expected based on chi-square analysis using a 95% confidence level (Table 4).

**Table 4.** Progeny analysis from self-pollinated *Zea mays* L. T0 events. Representative seed set and progeny data for self-pollinated single copy T0’s transformed with PHP79066 are shown

			Progeny analysis				
Genotype	Event	T0 seed set	Number of seeds sowed	Number of seeds germinated	Number homozygous	Number hemizygous	Number null
PH1V5T	1	264	32	32	7	16	9
PH1V5T	2	70	32	25	8	12	5
PH1V5T	3	336	32	29	5	17	7
PH1V5T	4	43	32	22	4	10	8
PHR03	1	335	32	26	8	12	6
PHR03	2	244	32	30	8	17	5

### Physical separation of somatic embryos resulted in increased plantlet formation

Early observations clearly showed high numbers of somatic embryos that started to grow on the scutellum of each *A. tumefaciens*-infected zygotic embryo (see Fig. 3*A*–*D*). However, as these somatic embryos continued to develop and germinate, typically only 1–2 plantlets would develop robustly from the scutellar surface of the originally transformed zygotic embryo (see Fig. 4*E*, *F*). This suggested that embryo competition between these crowded embryos might be occurring, so the separation of these small developing somatic embryos was attempted to evaluate their subsequent development.

**Figure 7.**
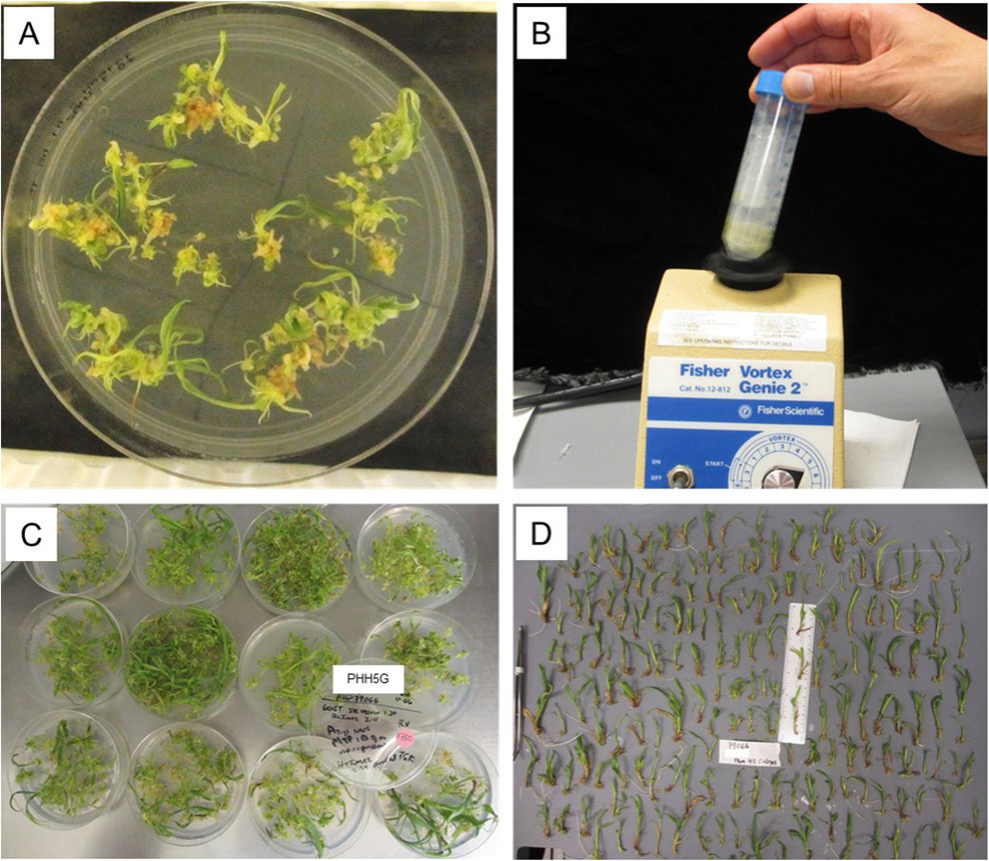
Using vortexing to separate germinating *Zea mays* L. somatic embryos. At 20 d post *Agrobacterium tumefaciens* infection, all the developing tissue (*A*) was transferred to liquid and vortexed (*B*). The tissue was distributed back onto germination medium. The number of recovered T0 plantlets was high (*C*) and from a total of 45 originally transformed zygotic embryos, a total of over 380 independent transgenic plantlets were recovered (*D*)

To test whether this suspected somatic embryo competition could be mitigated, the population of somatic embryos growing on 45 originally infected immature embryos from inbred PHH5G were collected after 2 wk on maturation medium and vortexed in liquid medium to separate the somatic embryos. The maturing somatic embryos were then re-plated onto germination medium. This resulted in over 380 plants being recovered from the originally transformed 45 starting embryos. qPCR analysis of a subset of 152 individual plantlets indicated that the individual analyzed plantlets were not clonal but instead were independent transgenic events, and of the 152 analyzed, 48 were identified as single copy plants resulting in a UQE% of greater than 100% (Fig. 7).

### Plant morphology and fertility

Transgenic T0 plants containing a single copy of the T-DNA from PHP79066 were grown to fertility in the greenhouse. These plants were indistinguishable from wild-type inbred plants and were fully both male and female fertile. Segregating T1 progeny from these plants had moderate to high germination rates with uniform robust morphology. In some events, homozygous plants were slightly shorter in stature than nulls but fully fertile, suggesting that some pleiotropy might still have been occurring. While single-copy plants were phenotypically normal, abnormal plants from multicopy events derived from particle gun transformations have also been observed.

## Discussion

Lowe *et al.* (2016) previously demonstrated that the maize morphogenic regulators *Bbm* and *Wus2* can be used to stimulate growth of embryogenic callus from immature embryos and normally recalcitrant tissues such as leaves or explants derived from mature seeds. Constitutive expression of *Bbm* and *Wus2* has severe pleiotropic effects such as wrinkled leaves (WUS, see Lenhard *et al.* 2002), sterility, and poorly functioning, thickened roots (Boutelier *et al.* 2002). To avoid these problems, an efficient excision system using LoxP sites and desiccation-induced CRE excision was developed (Lowe *et al.* 2016). As an alternative to CRE-mediated excision, a search to identify regulatory elements that would limit expression to transformation targets, specifically, scutella, and young leaves was undertaken. By eliminating expression in roots, meristems, and reproductive tissue, it was hypothesized that controlled expression could allow for the development of more normal plants while enhancing transformation in alternative targets such as mature seeds.

It was also hypothesized that by restricting expression to normally regenerable tissues, the transformation process would be enhanced by limiting the growth stimulation of the morphogenic genes to pluripotent cells. Since *Bbm* expression causes root abnormalities, a search for promoters that were not expressed in roots was undertaken. Furthermore, since in previous methods, cultures progressed through a process of embryogenic callus formation, it would be desirable for expression of *Bbm* to also occur in embryos and embryogenic callus. Expression in young leaves is also desirable since leaf primordia from mature seeds were demonstrated to have good transformation potential (Lowe *et al.* 2016). For that reason, a genome wide search was undertaken to find candidate promoters to drive this specific pattern of expression. Based on the desired selection criteria, promoters for these genes were cloned and evaluated. From a survey of over 65,000 gene models in a proprietary database, only one gene, a phospholipid transferase protein gene (*Zm-PLTP*) met the criteria perfectly. In addition, the non-Illumina®-derived ESTs for this gene were from libraries prepared from either zygotic embryos or from embryogenic callus, confirming the desired expression pattern. *Zm-PLTP*_*pro*_ was chosen to drive *Bbm* expression for two reasons. First, as described above, it was desired to prevent the expression of *Bbm* in root tissues, and second, such expression patterns might represent a viable alternative to excising *Bbm* and *Wus2* in order to avoid deleterious pleiotropic effects in maize roots and reproductive structures, often leading to either lethality or sterility.

Expression levels for both the *Ubiquitin* and *PLTP* transcripts were similar based on RNA obtained from whole embryo extracts (Fig. 2). However, since the *Zm-PLTP*_*pro*_::*ZsGREEN* expression was confined to the outer layers of the scutellum (Fig. 3*D*), it is likely that *PLTP* expression was much higher than *Ubiquitin* in the localized region of the scutellum epithelial layer. Expression of *ZsGREEN* from rescued *Zm-PLTP*_*pro*_::*ZsGREEN* embryos increased after placement on culture medium suggesting that culture stress also increased expression but was later down regulated once embryo fate was established. This is consistent with data demonstrating that *PLTP* expression was elevated under drought and cold stress conditions. Transcript profiling of developing somatic embryos showed very weak expression of *PLTP* transcripts during early stages of embryo development (data not shown), but at later stages of embryo development expression was stronger (Fig. 2).

The first vectors with the new *PLTP* promoter driving expression of *Bbm* were not only designed to test the impact of this expression cassette on transformation frequencies but also to assess later pleiotropic effects on plant development (i.e., with no recombinase-mediated excision before germination). When immature embryos were transformed with *Zm-PLTP*_*pro*_::*Bbm*, the transgenic response was both rapid and consistent. After *A. tumefaciens* infection with this expression cassette in combination with either an *Zm-Axig1*_*pro*_::*Wus2* or *Nos*_*pro*_::*Wus2* cassette, somatic embryos could be observed just 2 d later arising from the scutellar epithelia layer. This response was auxin-dependent which is consistent with auxins almost always being required for the initiation of embryogenic cultures in maize (Samaj *et al.* 2003) and embryogenic cultures in general (Zimmerman 1993; von Arnold *et al.* 2002). Without auxins, the immature zygotic embryo explants immediately germinate, and no somatic embryos are formed. Close to 100% of the infected embryos were covered with these somatic embryos and this response appeared to be genotype-independent. To date, this method has been tested in more than 22 of Pioneer’s current production inbred lines as well as the public inbred lines B73, Mo17, and FFMM line A (McCaw *et al.* 2016) and all have been responsive. These early somatic embryos were well formed and similar in appearance to those observed in *Dactylis* (Conger *et al.* 1983) and *Oryza* (Jones and Rost, 1989).

The rapid *de novo* somatic embryo development observed within the first week closely recapitulated zygotic embryo development as described by Kiesselbach (1949). Analogous to zygotic embryos, the somatic embryos observed grew rapidly and mitotic figures were readily observed in cross-section. Embryos containing approximately 750–800 cells were observed within 4 d of *A. tumefaciens* infection, again emulating the 4-d-old zygotic embryos observed by Kiesselback (1949). After 7 d, the somatic embryo development became more aberrant and typical of previous description by Van Lammeren (1988) of somatic embryos in maize, who also observed rescued zygotic embryos developing abnormally when placed in culture, possibly due to the lack of physical restraints during growth.

Single transformed immature zygotic embryos often produced dozens of somatic embryos on the surface of the scutellum. However, only a fraction of these somatic embryos ultimately germinated (compare the number of fluorescent embryos in Fig. 4*B-C* with the number of plants produced from each of the originally transformed immature embryos in Fig. 4*F*). Part of this attrition could be due to the fact that no effort was taken to orientate these somatic embryos on the culture medium. Matthys-Rochon *et al*. (1998) found that embryo germination was greatly improved when the developing meristem was not in contact with the culture medium after the transition stage. Competition between developing embryos presents another possible explanation for somatic embryo attrition during development and germination. In some genotypes, such as PHH5G, the somatic embryos are loosely connected and can be easily separated early in development, resulting in much higher numbers of recovered individual transgenic plants. This observation suggested that somatic embryo attrition may be influenced by competition or inhibition by unknown factors. Similarly, when polyembryony occurs in nature it is common to have single dominant embryos (Filonova *et al.* 2002).

It appears that these somatic embryos are of single cell origin for the following reasons. First, evidence that favored a single cell origin include (i) early somatic embryos that formed on the surface of the scutellum uniformly expressed fluorescent proteins and were separated by non-fluorescing zygotic scutellar surface (Fig. 4*C*), (ii) chimeric plants were not observed, and (iii) for individual transgenic plants analyzed by SbS™, the junctions between the integrated T-DNA and the genome were unique for every plant (Fig. 6). Secondly, observations that were consistent with a single cell origin for the somatic embryos include (i) microscopy suggested single cell origin based on the fact that individual early embryos were only observed growing from the zygotic scutellar surface, (ii) the growth of embryos was rapidly induced by the morphogenic genes and the T-DNA was delivered into single cells, and (iii) unlike during organogenesis, the embryos were never connected to the underlying tissue via vascular connections.

Embryo development and germination continued at a rapid pace and using these new expression constructs transgenic plants were obtained from many inbred lines in just 24–30 d. This is roughly the same time it takes to obtain plants from rescued zygotic embryos. Using this method, plants can be sent to the greenhouse in less than half the time of the traditional process. Using these simple non-excision vectors, transgenic T0 plants were routinely recovered at frequencies ranging between 9 and 224%, which varied between inbred lines and between batches of immature embryos from separate ears for the same inbred (transformation frequencies in Tables 2 and 3). Variability in transformation results between genetic backgrounds (i.e., different inbred lines or different varieties in other crops) has historically been a common observation in the field of plant biotechnology, and while this new method has worked in all the maize inbred lines tested to date, the influence of genetic background on the transformation process has not been eliminated. In addition, this method has not eliminated the ear-to-ear variability that is typically observed even within the same maize inbred where plants have been grown side-by-side in the greenhouse. This has typically been attributed to some physiological difference in the plants, but more specific information has remained elusive.

By comparison, traditional callus-based transformation protocols are labor-intensive and calluses must be bulked up for many weeks prior to regeneration and maintained long enough with selective pressure to eliminate escapes. During this prolonged selection period, care must be taken to preserve event identity with calluses derived from individual zygotic embryos maintained on separate plates to insure event purity. In contrast, with this new callus-free method, every plant was a separate event and numerous events were maintained on a single plate without the need for physical separation of events.

While *Zm-PLTP*_*pro*_::*Bbm* can be used with either *Nos*_*pro*_::*Wus2* or *Zm-Axig1*_*pro*_::*Wus2* to induce rapid somatic embryos, from previous experiments T1 seed containing *Nos*::*Wus2* have germination problems. These germination problems can be overcome by using the maize auxin-inducible *Zm-Axig1*_*pro*_ to drive expression of *Wus2*, again alleviating the need for gene excision. Surprisingly, by using the *Zm-PLTP*_*pro*_ and restricting *Bbm* expression to silks, leaf cork, and leaf subsidiary cells the plants were indistinguishable from wild-type inbred plants and were both male and female fertile. Thus using the combination of *Zm-Axig1*_*pro*_::*Wus*2 with *Zm-PLTP*_*pro*_::*Bbm* normal plants were recovered without excision.

Using imazapyr selection with the double mutant *Hra* gene, it was found that all the plants that regenerated on selection were transformed. Surprisingly, other traditional maize selective agents such as bialaphos (for the *moPAT* gene) and mannose (for the *PMI* gene) work poorly leading to a high percentage of escapes. Possibly conventional callus tolerates a slow kill during selection. In contrast, direct embryo formation requires a more stringent, fast-acting selection due to the rapid rate of cell division and direct germination. Compared to conventional callus-based selection in maize, the formation of single somatic embryos that rapidly produce plants presents new challenges for selection of transgenic events and further development of these methods is required.

The ability to provide fertile plants without excision has significant implications for genome editing. Since edits are not linked to the integration of the morphogenic genes, segregation can be used to separate the edits from the transgenes in the following generation, minimizing the importance of obtaining single-copy transformants. However, presently, routine screening for copy number is done and only single-copy plants are sent to the greenhouse. It is possible and likely that high copy number will have an effect on plant phenotype and fertility.

All transformation experiments were performed using a ternary vector system recently described by Anand *et al.* (2017a, b). The transformation results are facilitated by the *A. tumefaciens* strain and the presence of an accessory plasmid pPHP71539 (GenBank accession# MF788073). The results presented here utilized an auxotrophic LBA4404 THY- harboring the accessory plasmid and the binary plasmid containing an extra set of virulence genes from Bo542 (VirB1-E3). This is the preferred combination of *A. tumefaciens* and accessory plasmid for this method.

## Conclusions

The method described here is believed to be genotype-independent, fast, and simple. Provided one has access to a source of immature embryos, this method should enable any laboratory with tissue culture expertise to produce transgenic maize plants from any genotype. Using variations of this method (using the same basic components in different configurations and delivery methods), DuPont Pioneer has produced tens of thousands of independently transformed plants. Currently, variations of this method are being used for CRISPR/CAS9 genome editing and these methods have worked in every genotype tested to date (currently 22 pioneer and three public inbred lines). The true test of genotype independence will require testing in other labs with access to unique germplasm.

## References

[CR1] An G (1986). Development of plant promoter expression vectors and their use for analysis of differential activity of nopaline synthase promoter in transformed tobacco cells. Plant Physiol.

[CR2] An G, Mitra A, Choi HK, Costa MA, An K, Thornburg RW, Ryan CA (1989) Functional analysis of the 3' control region of the potato wound-inducible proteinase inhibitor II gene. Plant Cell 1:115–22 10.1105/tpc.1.1.115 PMC159742 2535459

[CR3] Anand A, Arling M, Da Silva A, Gordon-Kamm WJ, Hastings CE, Hoerster GM, Klein TM, La Rota CM, Lowe KS, Tiwari SB, Wang N, Wu XE (2017a) Methods and compositions for rapid plant transformation. Patent Number US20170121722A1

[CR4] Anand A, Bass SH, Cho H-J, Klein TM, Lassner M, McBride KE (2017b) Methods and compositions of improved plant transformation. Patent Number WO 2017/078836

[CR5] Boutelier K, Offringa R, Sharma VK, Keift H, Ouellet T, Zhang L, Hattori J, Liu C-M, Lammeren AMM, Miki BLA, Custers JBM, van Lookeren Campagne MM (2002). Ectopic expression of baby boom triggers a conversion from vegetative to embryonic growth. Plant Cell.

[CR6] Bhyri P, Khrishnamurthy N, Narayanan E, Nott A, Sarangi RR (2014) Novel plant terminator sequences. Patent Number US2014/0130205

[CR7] Busk PK, Jensen AB, Pagès M (1997). Regulatory elements *in vivo* in the promoter of the abscisic acid responsive gene *rab17* from maize. Plant J.

[CR8] Christensen AH, Sharrock RA, Quail PH (1992). Maize polyubiquitin genes: structure, thermal perturbation of expression and transcript splicing, and promoter activity following transfer to protoplasts by electroporation. Plant Mol Biol.

[CR9] Conger BV, Hanning GE, Gray DJ, McDaniel JK (1983). Direct *embryogenesis* from mesophyll cells of *Orchardgrass*. Science.

[CR10] Filonova LH, von Arnold S, Daniel G, Bozhkov PV (2002). Programmed cell death eliminates all but one embryo in a polyembryonic plant seed. Cell Death Diff.

[CR11] Garnaat C, Lowe K, Roth B (2002) *Zm-AXIG1*-specific polynucleotides and methods of use. Patent Number WO2002006499

[CR12] Gatz C, Frohberg C, Wendenburg R (1992). Stringent repression and homogeneous de-repression by tetracycline of a modified CaMV 35S promoter in intact transgenic tobacco plants. Plant J.

[CR13] Gordon-Kamm WJ, Helentjaris TG, Lowe KS, Shen B, Tarczynski MC, Zheng P (2005) Ap2 domain transcription factor Odp2 (ovule development protein 2) and methods of use. Patent Number WO2005075655

[CR14] Green CE, Phillips RL (1975). Plant regeneration from tissue cultures of maize. Crop Sci.

[CR15] Green JM, Hale T, Pagano MA, Andreassi JL, Gutteridge SA (2009). Response of 98140 corn with gat4621 and hra transgenes to glyphosate and ALS-inhibiting herbicides. Weed Sci.

[CR16] Guo J, Hammar M, Öberg L, Padmanabhuni SS, Bjäreland M, Dalevi D (2013). Combining evidence of preferential gene-tissue relationships from multiple sources. PLoS One.

[CR17] Ishida Y, Satto H, Ohta S, Hiei Y, Komari T, Kumashiro T (1996). High efficiency transformation of maize (Zea mays L.) mediated by *Agrobacterium tumefaciens*. Nat Biotechnol.

[CR18] Ishida Y, Hiei Y, Komari T (2007). *Agrobacterium*-mediated transformation of maize. Nature protoc.

[CR19] Jayne S, Barbour E, Meyer T (2000) Methods for improving transformation efficiency Patent Number US6096947

[CR20] Ji Q, Xu X, Wang K (2013). Genetic transformation of major cereal crops. Int J Dev Biol.

[CR21] Jones TJ, Rost TL (1989). The developmental anatomy and ultrastructure of somatic embryos from rice (*Oryza sativa L*.) scutellum epithelial cells. Bot Gaz.

[CR22] Kadota K, Konishi T, Shimizu K (2007). Evaluation of two outlier-detection-based methods for detecting tissue-selective genes from microarray data. Gene Regul Syst Bio.

[CR23] Kadota K, Nishimura SI, Bono H, Nakamura S, Hayashizaki Y, Okazaki Y, Takahashi K (2003). Detection of genes with tissue-specific expression patterns using Akaike’s information criterion (AIC) procedure. Physiol Genomics.

[CR24] Kadota K, Ye J, Nakai Y, Terada T, Shimizu K (2006). ROKU: a novel method for identification of tissue-specific genes. BMC Bioinformatics.

[CR25] Kalla R, Shimamoto K, Potter R, Nielsen PS (1994). The promoter of the barley aleurone-specific gene encoding a putative 7 kDa lipid transfer protein confers aleurone cell-specific expression in transgenic rice. Plant J.

[CR26] Kiesselbach TA (1949). The structure and reproduction of corn.

[CR27] Lenhard M, Jurgens G, Laux T (2002). The WUSCHEL and SHOOTMERISTEMLESS genes fulfil complementary roles in Arabidopsis shoot meristem regulation. Development.

[CR28] Li B, Dewey CN (2011). RSEM: accurate transcript quantification from RNA-Seq1 data with or without a reference genome. BMC Bioinformatics.

[CR29] Lowe KS, Cahoon RE, Scelonge CJ, Tao Y, Gordon-Kamm WJ, Bruce WB, Newman LJ (2007) Wuschel (WUS) gene homologs. Patent Number US7256322B2

[CR30] Lowe K, Wu E, Wang N, Hoerster G, Hastings C, Cho M-J, Scelonge C, Lenderts B, Chamberlin M, Cushatt J, Wang L, Ryan L, Khan T, Chow-Yiu JJ, Hua W, Yu M, Bahn J, Bao Z, Brink K, Igo E, Rudrappa B, Shamseer PM, Bruce W, Newman L, Shen B, Zheng P, Bidney D, Falco C, Register J, Zhao Z-Y, Xu D, Jones T, Gordon-Kamm W (2016). Morphogenic regulators Baby boom and Wuschel improve monocot transformation. Plant Cell.

[CR31] Matthys-Rochon E, Piola F, Le Deunff E, Mol R, Dumas C (1998). *In vitro* development of maize immature embryos: a tool for embryogenesis analysis. J Exp Bot.

[CR32] Matz MV, Fradkov AF, Labas YA, Savitsky AP, Zaraisky AG, Markelov ML, Lukyanov SA (1999). Fluorescent proteins from nonbioluminescent Anthozoa species. Nature Biotech.

[CR33] McCaw ME, Wallace JG, Albert PS, Buckler ES, Birchler JA (2016). Fast-flowering mini-maize: seed to seed in 60 days. Genetics.

[CR34] Negrotto D, Jolley M, Beer S, Wenck AR, Hansen G (2000). The use of phosphomannose–isomerase as a selectable marker to recover transgenic maize plants (*Zea mays* L.) via *Agrobacterium* transformation. Plant Cell Rep.

[CR35] Pujar A, Jaiswal P, Kellogg EA, Ilic K, Vincent L, Avraham S, McCouch S (2006). Whole-plant growth stage ontology for angiosperms and its application in plant biology. Plant Physiol.

[CR36] Randolph LF (1936). Developmental morphology of the caryopsis of maize. J Agric Res.

[CR37] Reed J, Privalle L, Powell ML, Meghji M, Dawson J, Dunder E, Suttie J, Wenck A, Launis K, Kramer C, Chang Y-F, Hansen G, Wright M (2001). Phosphomannose isomerase: an efficient selectable marker for plant transformation. In Vitro Cell Dev Biol-Plant.

[CR38] Ritchie SW, Hanway JJ, Benson GO (1986) How a corn plant develops. CES Special Report No. 48. Iowa State University, Ames, IA

[CR39] Samaj J, Baluska F, Pretova A, Volkman D (2003). Auxin deprivation induces a developmental switch in maize somatic embryogenesis involving redistribution of microtubules and actin filaments from endoplasmic to cortical cytoskeletal arrays. Plant Cell Rep.

[CR40] Van Lammeren AAM (1988). Observations on the structural development of immature maize embryos (*Zea mays* L.) during *in vitro* culture in the presence or absence of 2,4-D. Acta Bot Neerl.

[CR41] Von Arnold S, Sabala I, Bozhkov P, Dyachok J, Filonova L (2002). Developmental pathways of somatic embryogenesis. Plant Cell Tissue Organ Cult.

[CR42] Wang K (2005) Maize genetic transformation: *Agrobacterium*-mediated. In: Plant Transformation Facility. http://ptf.agron.iastate.edu/service/agromaize.aspx

[CR43] Wang K, Frame B, Ishida Y, Komari T (2009) Maize transformation. In: Bennetzen K, hake S (eds) Handbook of maize. Genetics and genomics. New York: Springer Science + Business Media, pp. 609–639

[CR44] Zastrow-Hayes GM, Lin H, Sigmund AL, Hoffman JL, Alarcon CM, Hayes KR, Richmond TA, Jedeloh JA, May GD, Beatty MK (2015) Southern-by-sequencing: a robust screening approach for molecular characterization of genetically modified crops. Plant Genome 8. 10.3835/plantgenome2014.08.0037 10.3835/plantgenome2014.08.0037 33228291

[CR45] Zhao Z-Y, Gu W, Cai T, Tagliani L, Hondred D, Bond D, Schroeder S, Rudert M, Pierce D (2002). High throughput genetic transformation mediated by *Agrobacterium tumefaciens* in maize. Mol Breed.

[CR46] Zimmerman JL (1993). Somatic embryogenesis: a model for early development in higher plants. Plant Cell.

